# A governance framework for blockchain-based manufacturing collaborative platform

**DOI:** 10.1016/j.fmre.2024.12.012

**Published:** 2025-06-05

**Authors:** Gengzhong Feng, Zhecheng Wang, Xi Zhao, Ningning Liu, Xiaomin Shi

**Affiliations:** a School of Management, Xi’an Jiaotong University, Xi'an 710049, China; b System Behavior & Management Laboratory of Xi’an Jiaotong University, Philosophy and Social Sciences Laboratory of the Ministry of Education, Xi'an 710049, China; c The Key Lab of the Ministry of Education for Process Management & Efficiency Engineering, Xi’an 710049, China; d School of Information Technology and Management, University of International Business and Economics, Beijing 100029, China

**Keywords:** Governance framework, Blockchain technology, Manufacturing collaborative platform, Platform governance, Distributed manufacturing, Autonomous organization

## Abstract

The traditional manufacturing collaborative platforms (MCPs) face challenges of weak trust and innovation inhibition in the transformation and upgrading of the global industrial chain. This paper investigates the blockchain-based manufacturing collaborative platforms (BMCPs) as a potential solution. This paper first analyzes the ecological characteristics of MCPs and BMCPs, and puts forward six governance challenges caused by the unique openness, uncertainty and complexity of BMCPs. Then, in order to offer comprehensive and adaptable guidelines for BMCP governance practices, we propose a governance framework with three layers (off-chain community, on-chain DAO, and on-chain contracts) and five dimensions (roles, incentives, communication, decision-making, and value evolution). Finally, based on the value evolution characteristics of BMCPs, we investigate how to apply the proposed governance framework during the start-up and optimization stages of BMCPs. Using member expansion and fault management as two-stage examples, the paper uses a UML sequence diagram to demonstrate how the governance framework works. This study provides a theoretical framework and practical guidance for creating a more transparent, intelligent and sustainable manufacturing ecosystem.

## Graphical abstract

**Figure:**
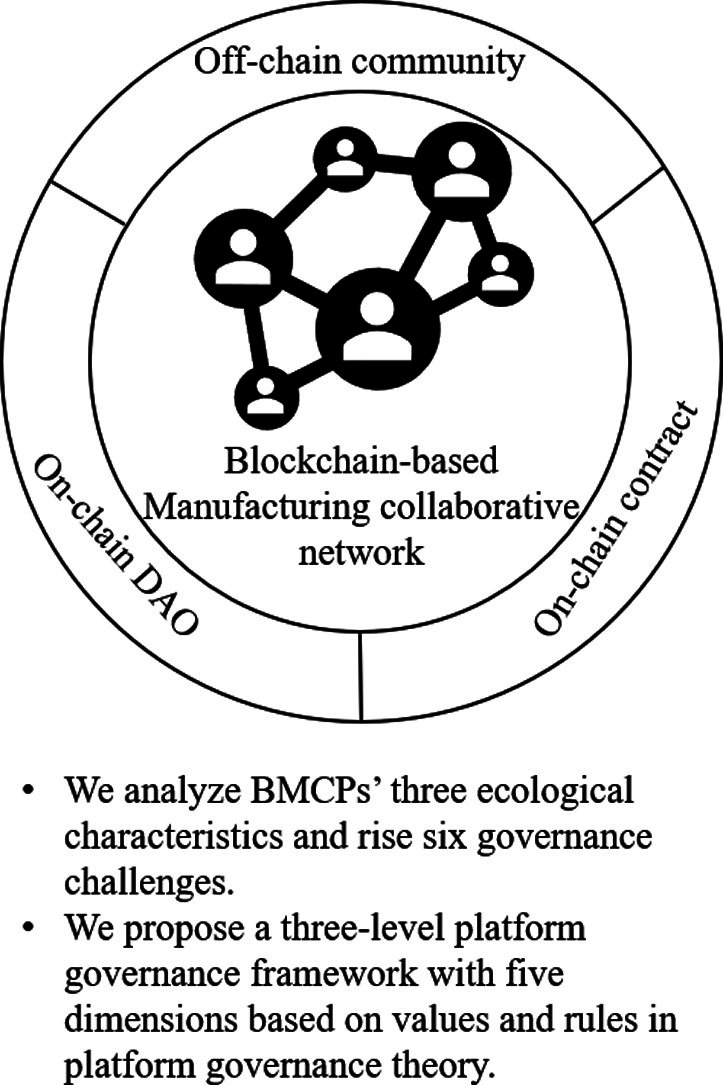

## 1 Introduction

The manufacturing industry is undergoing a significant transformation as a result of the platform economy, which is driving it in the direction of more efficiency, flexibility, and collaboration [1]. The Manufacturing Collaborative Platform (MCP), as the core driver of this transformation, represents an intensive and flexible production model. By employing standardized components and resource sharing, it strives to decrease the time from research and development to market and accelerate product creation cycles, all while successfully compressing costs and improving overall productivity [[2], [3], [4]]. These MCPs provide a holistic solution that covers the whole product lifecycle by optimizing not only the physical product design and production processes but also supply chain management [5], logistics coordination, after-sales services, and a host of other value-added services [6]. Businesses are embracing MCPs with great enthusiasm as a strategic decision to boost their competitiveness [7].

However, even though traditional MCPs have greatly increased production efficiency and resource utilization in the global manufacturing sector, a number of long-standing problems have surfaced with the growth of business scale and require immediate attention. The primary challenge is supply chains' growing complexity and unpredictability. Conventional information management techniques seriously impede cross-enterprise collaboration since they are opaque and quickly lead to the creation of data silos. This severely limits the platform's potential to create value and is especially problematic for data security and creativity. Furthermore, a constant stream of poor quality and an abundance of fake and inferior products undermine consumer trust and damage firms' reputations and positions in the marketplace [[8], [9], [10]]. According to a McKinsey analysis, significant operational interruptions lasting a month or more typically occur every 3.7 years in important manufacturing industries. Shorter disruptions are even more frequent, highlighting the vulnerability of the current production systems.^1^ Therefore, it is imperative to integrate emerging technologies to drive the comprehensive upgrade and transformation of MCPs, aiming to build a more robust, intelligent, and secure industrial ecosystem.

Given this, the introduction of blockchain technology presents an alternative to traditional MCPs, radically altering the manufacturing sector's governance and operational frameworks. As a decentralized and immutable digital ledger technology, blockchain can establish trust without the need for third-party intermediaries through its unique distributed consensus mechanism. In the manufacturing process, blockchain ensures that all parties in the supply chain can access and verify transaction data in real-time, enhancing information transparency and integrity [11]. Additionally, the smart contract functionality of blockchain allows for the automatic execution of complex transaction conditions, eliminating human errors in intermediary processes, significantly improving operational efficiency, and reducing costs. Compared to traditional manufacturing platforms, Blockchain-based Manufacturing Collaborative Platforms (BMCPs) demonstrate significant advantages in data security, transparency, and supply chain collaboration [12]. Under traditional MCPs, data often remains isolated in silos with no effective mechanisms for interconnectivity and sharing. BMCPs, however, achieve global data visibility through encryption and consensus algorithms, ensuring that all participants can make decisions based on the same authentic information. More importantly, blockchain technology enables BMCPs to establish a new governance framework that emphasizes decentralized power distribution. This is achieved through smart contracts and consensus mechanisms that facilitate automated governance, reducing reliance on centralized institutions and enhancing system resilience.

In today's highly interconnected business environment, platform governance has become a critical factor in determining the success of organizations. The essence of platform governance lies in establishing and implementing a set of guiding principles designed to regulate the behavior of participants within the platform, promote fair competition, protect user rights, and ensure sustainable development. In the manufacturing industry, the importance of platform governance is particularly pronounced. MCPs connect suppliers, manufacturers, distributors, and consumers, forming a complex ecosystem. Effective governance mechanisms can ensure the healthy operation of this ecosystem, including supply chain transparency, product quality control, and intellectual property protection. However, traditional MCP governance models are often hindered by information barriers, trust issues, and technological limitations, leading to inefficient governance that struggles to adapt to rapidly changing market demands.

The deep integration of blockchain technology is driving a fundamental transformation of MCPs, giving rise to BMCPs characterized by high transparency, traceability, and autonomous operations. BMCPs leverage decentralized strategies to significantly enhance supply chain efficiency and transparency, reduce transaction costs, and strengthen quality control and compliance management. This transformation reshapes the traditional openness, complexity, and uncertainty of MCPs, particularly by forming a complex and dynamic network of interactions among diverse stakeholders across different fields and levels. This shift not only presents significant challenges to traditional governance frameworks but also drives the governance focus from centralization to decentralization, emphasizing the importance of collaboration quality and value co-creation. In a decentralized context, the effectiveness of traditional control, incentive, and communication mechanisms is notably diminished, failing to meet the transparency and decentralization requirements of BMCPs. Therefore, there is an urgent need to transform governance paradigms to promote the free flow of information, ensure equal participation rights for all stakeholders, and maintain system stability and security. The aim is to create a new manufacturing ecosystem governance framework that fosters innovation while ensuring efficiency and fairness. In summary, blockchain technology not only catalyzes profound changes in the manufacturing ecosystem but also imposes new demands on governance methods, pushing for a shift towards decentralized governance to adapt to a more open, complex, and uncertain manufacturing environment, thereby achieving comprehensive optimization and upgrading of the manufacturing ecosystem.

At the forefront of Web3, Decentralized Autonomous Organizations (DAOs) are emerging as a disruptive governance model. DAOs utilize blockchain technology, particularly its distributed ledger features, to create a self-governing system without traditional centralized management. This innovative model stands in stark contrast to traditional platform governance, where decision-making power is typically concentrated in the hands of a few managers or board members, with limited information flow and susceptibility to internal biases or external pressures. In contrast, DAOs use smart contracts to automatically execute predefined rules, ensuring that all decision-making processes are open and transparent, granting every participant equal governance rights. This greatly enhances the democratization and efficiency of decision-making, which holds significant reference value for the governance of BMCPs.

However, current research primarily focuses on the theoretical exploration and technical details of blockchain technology, with limited attention given to its practical effects and impacts in manufacturing scenarios. Existing studies on platform ecosystems and governance mechanisms are predominantly centered on traditional centralized platforms, failing to adequately consider the profound changes brought by blockchain technology to platform governance. This oversight restricts the effective implementation and widespread adoption of Blockchain-based Manufacturing Collaboration Platforms (BMCPs). In light of this, the present study aims to fill this knowledge gap by addressing three core research questions:

RQ1: How does the ecosystem of BMCPs differ from that of MCPs, and what governance challenges does this transformation present?

RQ2: How should BMCPs construct a governance framework that meets their unique needs?

RQ3: How can BMCPs effectively apply this framework in practice to manage the dynamic changes in the platform ecosystem over time?

By addressing these issues, this paper makes contributions in both theoretical and practical aspects: On the one hand, the paper constructs a BMCP governance framework that encompasses five dimensions—roles, incentives, communication, decision-making, and value evolution—across three layers: the off-chain community layer, the on-chain DAO layer, and the on-chain contract layer. This framework systematically elucidates the design of governance mechanisms for BMCPs by analyzing the evolution of BMCP ecosystem characteristics and the challenges they face. This work not only enriches platform governance theory but also provides theoretical guidance for the application of blockchain technology in manufacturing platform governance. On the other hand, the BMCP governance framework proposed in this paper holds significant practical implications for the manufacturing industry. It offers practical governance tools for manufacturing platforms, assisting platform managers in addressing challenges within the BMCP ecosystem and ensuring the healthy operation and development of the platform. By providing dynamic governance strategies, the framework also offers strategic guidance for the governance needs at different stages of BMCP development, facilitating continuous optimization and upgrading of the platform, and ultimately promoting innovation and collaboration within the manufacturing ecosystem.

The remainder of this paper is organized as follows. Section 2 reviews the relevant literature on traditional platform governance (MCP governance) and blockchain-based platform governance (BMCP governance). Section 3 provides an in-depth analysis of the characteristics and governance challenges of the BMCP ecosystem. Section 4 proposes a comprehensive governance framework designed to address the multi-dimensional governance issues faced by BMCPs. Section 5 discusses the dynamic governance strategies BMCPs should adopt at different stages of development to adapt to the evolving ecosystem. Finally, Section 6 concludes the study and outlines future research directions.

## 2 Literature review

The governance of complex ecosystems is a significant challenge for MCP operations. Blockchain technology can provide an integrated solution for MCP, promoting the development of the BMCP research field. BMCP not only enhances the flexibility and reliability of MCP at the business level but also improves the openness and adaptability of MCP at the governance level. In this context, the literature flow related to this study can be divided into two groups: (1) MCP and platform governance; and (2) BMCP and blockchain governance.

### 2.1 MCP and platform governance

Digitalization is becoming one of the crucial drivers of innovation in the manufacturing industry, compelling manufacturers to connect the physical and digital worlds [13]. Digital technologies not only facilitate the servitization transformation of the manufacturing industry [14] but also give rise to digital platforms [1,15]. With the advancement of information technology and the expansion of industrial alliances, various types of digital platforms have emerged in the manufacturing industry, such as manufacturing network platforms [16], cloud manufacturing platforms [17], and industrial internet platforms [18]. Studies have shown that manufacturers can use these platforms to leverage data from various objects, devices, and scenarios to improve productivity and profitability [[19], [20], [21], [22]], creating higher value than traditional manufacturing models [23]. Additionally, platforms enable the sharing of manufacturing resources as services in both the physical and digital worlds, promoting cross-organizational resource collaboration [24]. Specifically, we collectively refer to these platforms that enable manufacturing resource sharing and provide service products as MCP.

The complex operating environment makes MCP characteristics and challenges different from other types of digital platforms. Jovanovic et al. [25] categorized platform prototypes into product platforms, supply chain platforms, and platform ecosystems, identifying unique innovation mechanisms and governance models for different types of platforms. Valilai and Houshmand [26] found that the sensitivity of shared information, the effectiveness of management tools, and information utilization all affect the efficiency of collaboration among members. Li et al. [24] analyzed the evolution process and environmental challenges of platforms, proposing a multi-party collaboration framework to guide the construction of platforms by manufacturing organizations. Kapoor et al. [27] developed an analytical framework for platform ecosystems using a multi-case study approach, considering four dimensions: technological core, key participants, structural boundaries, and servitization tasks. Moreover, MCP is constrained by technological and governance frameworks, where platform complementors have limited power and lack a foundation of trust, hindering their willingness to participate and collaborate [28]. This leads to the platform gradually transforming into a supply chain structure during operation, thereby losing the advantage of ecosystem complementarity, which is a major reason for the obstacles in the application and promotion of MCP [29]. To achieve value creation through the establishment of collaborative platforms, manufacturing organizations need to innovate in both technological and governance frameworks.

Platform governance refers to establishing behavioral standards applicable to all participants within a platform ecosystem [30], aiming to balance the personalized needs of different participants and achieve effective coordination of the platform ecosystem in a standardized manner [31]. According to the theory of platform ecosystem governance, platform governance can be divided into two components: rules and values [32]. These rules define the rights and responsibilities of platform members. Standardized resources within a platform ecosystem include key technological resources [33] and marketing resources [34], among others. Given the limited nature of platform resources, the core of platform governance is the rational allocation of resources that platform members can use. For example, rules typically include collaborator management, defining the resources available to participants in different roles within the platform [35]. The second component of governance is value, which describes the key objectives and collaborative spirit of the platform ecosystem [36]. Value is usually created by platform owners with specific goals and then realized collectively by platform participants. This value is often reflected in the platform's cooperative norms and value orientation [32]. Current research on platform governance includes the design and optimization of mechanisms, such as governing platform ecosystems from the dimensions of collaboration, resources, control, and market [37]; the mechanism by which leaders change platform rules to influence participants' behavior [38]; and the impact of macro culture and collective sanctions on platform user engagement [39]. With the development of information technology, the platform ecosystem and its governance mechanism are constantly evolving. However, there is a lack of research focusing on MCP governance mechanisms in the context of new technological environments.

### 2.2 BMCP and blockchain governance

With the paradigm shift in manufacturing from mass production to on-demand, personalized, customer-oriented, and knowledge-based production, engineers must innovate production network models. Decentralized manufacturing (DM) and autonomous manufacturing (AM) have become the new trends guiding MCP construction [40,41]. DM emphasizes the platform's coordination of geographically dispersed resources to achieve barrier-free cooperation among members. AM attaches importance to the transformation of platform manufacturing systems from automation to autonomy and realizes the flexibility of business collaboration. From the perspective of technological evolution path, blockchain and DAO become an important force in promoting the development of MCP [42]. Blockchain technology is a decentralized distributed ledger proposed in the white paper published by Satoshi Nakamoto in 2008 [43], which has the characteristics of decentralization, openness and transparency, invariability, and traceability. Therefore, this technology is a potential solution to the ecological challenge of MCP. For example, a blockchain-based decentralized digital payment system that can be automatically executed without the intervention of a trusted third party helps MCP to provide personalized services and improve the efficiency of information exchange [44]. Therefore, BMCP has been widely used in the manufacturing industry. At present, as a new platform mode, BMCP platform architecture has not formed a unified standard. We summarize the platform design under different scenarios in the literature (Table 1). Compared with MCP, BMCP has advantages in user participation, service collaboration, data sharing and knowledge innovation.

**Table 1:** **BMCP framework**.

Platform name	Architecture	Core function	Application	Feature
Collaborative platform [45]	Raw data layer, fog computing layer, big data analysis layer, knowledge layer, application layer, blockchain network	Customer feedback, manufacturing systems, design systems, big data storage and analysis	The service covers the whole process of user feedback from design to manufacturing, enabling direct communication between designers, manufacturers and customers	The platform is customer-centric for product development, combined with machine learning algorithms for clustering customer views
BPIIoT [46]	Device layer, network layer, service layer, application layer, on-chain network, off-chain network	Secure multi-party computing, rights management, intelligent predictive maintenance, device status data sharing	Realize the decentralized and trusted network interaction of devices in smart factories, open up the data channel between the industrial chain and the Internet of Things devices, and establish the industrial Internet of Things ecosystem	The platform combines on-chain and off-chain technologies to form a lightweight architecture that reduces network load and latency and provides on-demand service agreements through smart contracts
Block-SC [12]	/	Solver allocation, service composition, consensus mechanism, solver reward mechanism	Distributed technology is used to realize complex service combination calculations of a large number of heterogeneous resources in cloud manufacturing to meet the service demand of dynamic parameter changes	Through the design of mining solution, consensus, incentive and other mechanisms, the service composition task is decentralized crowdsourced management through the platform
FabRec [47]	Participant layer, block and data load layer, consensus algorithm layer, device layer, smart contract layer, node data exchange layer	Block data structure, device data model, participant relationship contract, IT integrated blockchain manager	Process multi-party manufacturing information in a decentralized manner, using smart contracts to establish paperless contracts between decentralized network participants	By distinguishing the types of smart contracts and data models that the platform needs to build, an intelligent manufacturing platform supporting designer interaction with equipment is established
BcDTCP [48]	Infrastructure layer, professional field layer, application layer, display layer	Digital twins, knowledge network graphs, smart contracts for business flows	The heterogeneous social manufacturing resources are integrated with distributed execution networks to solve the dependence on central agencies in social manufacturing	A timed color Petri Net-based workflow formulates collaboration logic into smart contracts executed on the blockchain
BCmfg [49]	Resource layer, perception layer, manufacturing service provider layer, infrastructure layer, application layer	On-demand security services, secure data exchange, Dual-mode execution of on-cloud/off-cloud services	Develop a cloud manufacturing platform with high security and scalability through the blockchain technology architecture	The overall architecture of the distributed manufacturing service platform is designed as a solution to the trust problem of centralized network and third-party operation in cloud manufacturing

Blockchain is not only a reliable platform infrastructure at the technical level, but its decentralized consensus and automatic execution features also assist in managing multi-role participants at the governance level. Blockchain governance has gradually become an emerging research field. Pelt et al. [50] define blockchain governance as “the means of achieving stakeholder direction, control, and coordination in a specific blockchain project context, where they collectively contribute to the project,” and divide their framework into three layers: off-chain community, off-chain developers, and on-chain protocols. Rikken et al. [51] divide the term into four layers: infrastructure, applications, companies, and institutions, and analyze it from four themes: consensus, incentives, information flow, and governance structure. The decentralized and open governance model based on blockchain in distributed platforms has also become one of the unique attractions [52]. Firstly, the business coordination process of BMCP does not require third-party coordination, i.e., the control rights can be shared among independent entities, avoiding the power concentration that transforms the platform ecology into a supply chain structure [29,53]. Incorporating voting, collaborative work, and self-organizing democratic principles into the platform governance promotes the entry of new complementary players and protects them from the risks of unilateral rule changes by the platform [54]. Secondly, blockchain technology operates in a scalable, self-organizing system [55]. On the one hand, smart contracts overcome the limitations of unpredictable human decision-making and low information processing speed [56]. On the other hand, blockchain can reduce information asymmetry and opportunistic behavior between participants through machine automation [57]; for example, Walmart uses blockchain to solve traceability problems in the supply chain [58], and a Brazilian software company uses blockchain to share complex data for reliable state monitoring [59].

In particular, the DAO based on blockchain technology provides a basis for the organizational structure design of platform governance. Compared with traditional contract and relationship governance, DAO governance represents another independent rule system [10,60]. Daos can govern the BMCP ecosystem without a hierarchical governance structure [61], reducing governance costs. For example, through the automatic execution of on-chain smart contracts, platform rules can function without reliance on contract and relationship governance [62,63], which makes blockchain technology a better governance tool than other IT solutions such as EDI [64]. Dursun and Üstündağ [65] distinguish the blockchain-based governance model into on-chain governance and off-chain governance, in which off-chain governance is similar to the operation of direct democracy in social governance, and is based on community interaction to reach a consensus on platform rule changes. The off-chain governance process is usually led by one platform participant, which excludes a large number of users who lack the expertise and financial capital to influence decisions, but the data stored off-chain makes the process opaque. Well-known blockchain project change proposals Bitcoin (BIP 2021) and Ethereum (EIP 2021) were done by this model. On-chain governance is based on the immutable nature of the blockchain, where participants reach a consensus on platform rules or ledger changes in the form of votes. The governance model is highly decentralized, runs faster, and the process is transparent and auditable. However, the rules recorded on the chain and the decisions executed are difficult to change, and the code is still written by bounded rational actors, leading to potential risk vulnerabilities in the on-chain governance model [60,66,67]. Different types of distributed platforms are suitable for different governance models, and semi-decentralized governance models are proved to be more efficient and reliable [[68], [69], [70]]. Considering that the decentralization of BMCP makes the development of platform ecology unpredictable, and multi-level events such as machines, equipment, enterprises, and markets will have an impact on platform ecology, we propose a governance framework that balances centralization and decentralization [29,71]. The governance framework proposed in this paper is a three-layer structure of off-chain community, on-chain DAO and on-chain contract, which can provide solutions for governance mechanism design and operation management in different development stages of BMCP.

## 3 From MCP to BMCP: Ecological characteristics evolution and governance challenges

**Fig. 1:**
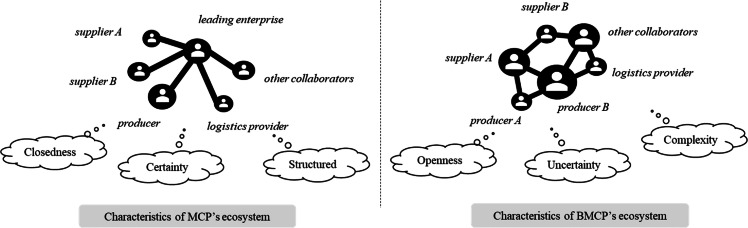
**Ecological characteristics of MCP & BMCP**.

A platform ecosystem is essentially a collaborative value network that requires a dynamic platform governance framework to support its operation. As shown in Fig. 1, the evolution of ecosystem characteristics from MCP to BMCP is from a closed, certain, and structured environment to a more open, uncertain, and complex one. This shift not only enhances the value creation potential of the platform ecosystem but also raises higher demands for platform governance. This chapter conducts an in-depth analysis of the evolving characteristics of the BMCP ecosystem, revealing six key challenges in platform governance. This lays the groundwork for subsequently developing a governance framework that aligns with the unique characteristics of the BMCP ecosystem.

### 3.1 Ecological characteristics of MCP & BMCP

From “closedness” to “openness”: In traditional MCP ecosystems, openness is often limited by the dominant position of core enterprises. The sharing of manufacturing resources and capabilities is restricted to a relatively closed circle, and the supply chain network built around the core enterprise does not achieve true social openness. In this model, the entry barriers for stakeholders are high, and the ability to meet personalized demands is limited by the resource allocation capabilities of the core enterprise, resulting in a semi-open, semi-closed form of collaboration. BMCP has fully socialized the supply and demand of manufacturing services through the deep integration of blockchain technology. Its openness is not only reflected in the sharing of resources and capabilities, but also in the democratization of platform governance [24]. Firstly, BMCP significantly lowers the entry barriers for platform stakeholders, providing standardized manufacturing resources and capabilities for various participants, thus promoting efficient resource allocation and sharing [72]. This openness not only meets the personalized needs of diverse stakeholders but also facilitates flexible and dynamic manufacturing alliances, enhancing the adaptability and innovation of the platform ecosystem. More importantly, BMCP introduces a decentralized consensus mechanism, granting stakeholders democratic participation in platform governance, thereby achieving unprecedented openness at the governance level. The benefits of this democratic governance mechanism include stimulating the long-term commitment of potential partners, driving manufacturing enterprises to continuously innovate business models and optimize production processes, ultimately building a truly open, collaborative, and innovative manufacturing ecosystem.

From “certainty” to “uncertainty”: Traditional MCPs rely on predefined rules, processes, and protocols to ensure that every link in the supply chain operates as planned, resulting in highly predictable collaboration processes. However, this certainty also means that MCP ecosystems have limited adaptability to change; when faced with unforeseen disruptions, the system's flexibility and responsiveness are compromised. In contrast, the uncertainty characteristic of BMCPs arises from the socialized nature of manufacturing service supply and demand. The development of the ecosystem is inherently unpredictable, and the decentralization enabled by blockchain technology amplifies this effect. In BMCPs, uncertainty primarily stems from disagreements and unpredictable behaviors during multi-party collaboration, such as differing interpretations of product quality standards between manufacturers and designers. This is particularly pronounced in long-chain manufacturing scenarios, where each stakeholder's actions can introduce uncertainty, spreading risk throughout the chain, increasing coordination costs, and impacting platform efficiency. However, blockchain technology's immutable data chains and smart contracts can effectively identify sources of uncertainty and govern them autonomously. Smart contracts can automatically adjust measures based on preset conditions and respond to changes in the supply chain in a timely manner, thereby reducing the negative impact of uncertainty. These mechanisms significantly improve the adaptability and resilience of the platform, allowing BMCP to maintain normal operation in uncertain environments.

From “structured” to “complexity”: Traditional MCPs are characterized by a high degree of regulation and organization concerning stakeholder participation, resource quality, and collaborative relationships. However, this excessive structuring also limits the platform's flexibility, making it difficult for MCPs to respond swiftly and adapt to dynamic market environments and diverse demands. The complexity of BMCPs arises from their departure from traditional structured models. In the BMCP ecosystem, the diversity of stakeholder motivations, varying resource quality, and dynamic changes in collaborative relationships create a highly complex network. The substitutability of different participants' capabilities and the differing expectations of stakeholders in the service provision process result in constantly shifting states of competition and cooperation, intensifying the complexity of interest conflicts. To address the complexity challenges of the BMCP ecosystem, DAOs based on blockchain smart contracts demonstrate unique advantages. DAOs' capabilities in fairness, transparency, and automated execution effectively coordinate the complex relationships among stakeholders. The automation of smart contracts ensures the fairness and transparency of transactions, reduces uncertainties associated with human intervention, and fosters trust. Through dynamic adjustment and execution of smart contracts, DAOs can flexibly address interest conflicts and resource allocation issues within the BMCP ecosystem.

### 3.2 BMCP governance challenges

The openness, uncertainty, and complexity of BMCP ecosystems present new demands and challenges for platform governance [[73], [74], [75], [76]]. Openness requires governance mechanisms that can accommodate broad participation and facilitate the free flow and sharing of resources. Uncertainty calls for more flexible and agile governance strategies to address unknown risks in the supply chain. Complexity challenges the precision of the governance framework, necessitating balance and coordination among diverse stakeholders. Consequently, this paper, problem-oriented, systematically identifies and summarizes the six core challenges facing BMCP governance:

1. What is the BMCP’s positioning? This challenge is closely related to openness and complexity. When BMCPs are positioned as a two-sided market to efficiently match supply and demand, their development goals often focus on profitability to ensure ongoing success in a competitive market. In such cases, platform governance tends to be led by core members, with centralized decision-making to enhance market responsiveness and economic efficiency. However, if BMCPs aim to provide infrastructure and value-added services for distributed collaboration, promoting the sustainable development of the entire ecosystem, the platform's positioning will reflect a more public-interest orientation. Here, governance might be led by government agencies or non-profit organizations, emphasizing fairness, transparency, and long-term value creation rather than short-term profit maximization. Therefore, the governance mechanism of BMCPs must be flexibly adjusted according to the specific application scenario, considering both the platform's commercial objectives and the ecosystem's complexity and openness to achieve dual enhancement of economic efficiency and social value. This positioning uncertainty requires a governance framework with sufficient flexibility and adaptability to balance the needs of different stakeholders.

2. What are the BMCP's main goals? Compared to traditional MCPs, BMCPs leverage blockchain technology to facilitate more efficient and transparent platform operations, empowering platform managers to achieve value co-creation and equitable distribution during the governance process [22]. This reflects the openness and complexity characteristics of BMCPs. However, the high data transparency introduced by blockchain technology also brings new uncertainties to real-time transactions among platform stakeholders, especially regarding the handling and protection of sensitive information. This interweaving of uncertainty and complexity complicates the risks faced by BMCP platform governance. Therefore, a core challenge for BMCP platform governance is managing the risks that accompany the advantages of data-driven approaches, ensuring data security and privacy protection, and achieving balanced value distribution and fair transactions among stakeholders. Achieving this goal requires a governance mechanism with high flexibility and adaptability to balance technological innovation and risk control.

3. Do the BMCP and stakeholders pursue the same values? This question is particularly significant in the governance of BMCPs and is closely related to the platform's openness and complexity. Stakeholders often share resources with the intention of leveraging others' high-quality resources to enhance their own value, which may include motives such as maintaining or gaining market dominance. In contrast, BMCP's core value goal focuses on maximizing overall benefits, including but not limited to improving the timeliness of manufacturing services, expanding the stakeholder network, enhancing user satisfaction, and optimizing the resource utilization efficiency of service providers [77]. This divergence in value goals suggests that stakeholder participation in platform decision-making may deviate from the goal of maximizing the platform's overall interests. In an open BMCP ecosystem, the diverse value pursuits of participants increase the complexity of decision-making mechanisms. The governance framework must not only promote efficient resource allocation but also ensure fairness and transparency in the decision-making process to align different interests and achieve synergy between platform and stakeholder value goals. Therefore, the design of decision-making mechanisms needs to balance efficiency and fairness to promote the healthy and sustainable development of the ecosystem.

4. Does BMCP governance need a hierarchical structure? This question is closely tied to the openness and uncertainty characteristics of BMCPs. Online platforms in their early stages often face unpredictable market conditions and operational risks. At this time, a hierarchical structure can provide more robust risk control and resource allocation mechanisms, helping the platform allocate assets more efficiently under limited resources. However, in a decentralized BMCP ecosystem, introducing a hierarchical structure may raise concerns about power centralization, contradicting the democratic governance principles of DAOs. The complexity of BMCPs requires governance mechanisms that ensure decision-making flexibility and responsiveness while maintaining ecosystem fairness and transparency. Consequently, the roles of stakeholders in BMCP governance, such as operators, manufacturers, and service providers, must dynamically adjust as the platform ecosystem evolves to balance the efficiency advantages of hierarchical structures with the fairness principles of decentralized governance. In BMCP governance practice, exploring how to introduce appropriate hierarchical structures to address uncertainty and complexity while maintaining platform openness and flexibility becomes another key challenge. This requires governance mechanisms that promote effective resource allocation and prevent power centralization, ensuring the rights and responsibilities of all participants are balanced as the platform develops.

5. Does BMCP governance require trust? In the BMCP ecosystem, collaboration among stakeholders has evolved from traditional relational and contractual trust to mechanism trust supported by smart contracts. However, the construction of a comprehensive trust mechanism is not achieved overnight, especially in the highly open and complex BMCP ecosystem, where establishing basic trust is an indispensable prerequisite. The uncertainty characteristic of BMCPs means that platform governance must consider the degree of trust dependence at different stages of development and how to gradually build and maintain this trust. In the early stages of the ecosystem, basic trust mechanisms are crucial for promoting collaboration. As the ecosystem matures, the automation and transparency of smart contracts play key roles in enhancing trust and reducing transaction costs. Therefore, the design of BMCP governance mechanisms must consider early-stage basic trust cultivation and later-stage smart contract trust mechanism enhancement to adapt to different stages of ecosystem development and ensure the healthy and stable operation of the platform. In BMCP governance practice, building and maintaining trust is an ongoing challenge that requires governance frameworks to respond flexibly to the openness and complexity of the ecosystem. By integrating smart contracts with basic trust, long-term cooperation and value co-creation among multiple stakeholders in the platform ecosystem can be promoted.

6. What are the effects on BMCP’s openness and stability? BMCP adheres to the principle of openness, providing equal services to all participants and allowing free entry and exit, thus creating a highly open collaborative ecosystem. In such an environment, key platform decisions must be achieved through collective consensus, highlighting the concept of decentralized governance. To ensure the effectiveness of the consensus mechanism, BMCP must design a scientific decision-making framework that supports decentralized governance within the collaborative network to balance openness and stability. The decision-making mechanism should be based on solid foundational rules, ensuring smooth operation on a small scale and ease of scalability. This means BMCP should follow principles that maintain a stable system, provide democratic decision-making tools, and reconcile the uncertainties brought by openness with the need for stable platform operation. Balancing openness and stability in BMCP governance is a core challenge, requiring a governance structure that promotes platform openness to attract more participants while ensuring the robustness of decision-making mechanisms to secure the long-term stability and healthy development of the platform ecosystem. By rationally designing decision-making mechanisms, BMCP can effectively manage the tension between openness and stability, creating a collaborative platform that is both open and stable.

Blockchain technology brings significant advantages to BMCP while also introducing new governance challenges. Its characteristics of openness, uncertainty, and complexity demand governance mechanisms with greater flexibility and adaptability to cope with the dynamic changes and diverse needs of the platform ecosystem. Facing six core challenges in BMCP governance, including platform positioning, value goal consistency, the necessity of hierarchical structures, trust mechanism construction, and the balance between openness and stability, it becomes crucial to analyze specific scenario characteristics and design governance solutions that match them. In the following chapter, this paper will propose a comprehensive governance framework aimed at helping BMCP effectively address the challenges encountered when applying blockchain technology in different scenarios, promoting the healthy, stable, and sustainable development of the platform ecosystem.

## 4 BMCP governance framework design

**Fig. 2:**
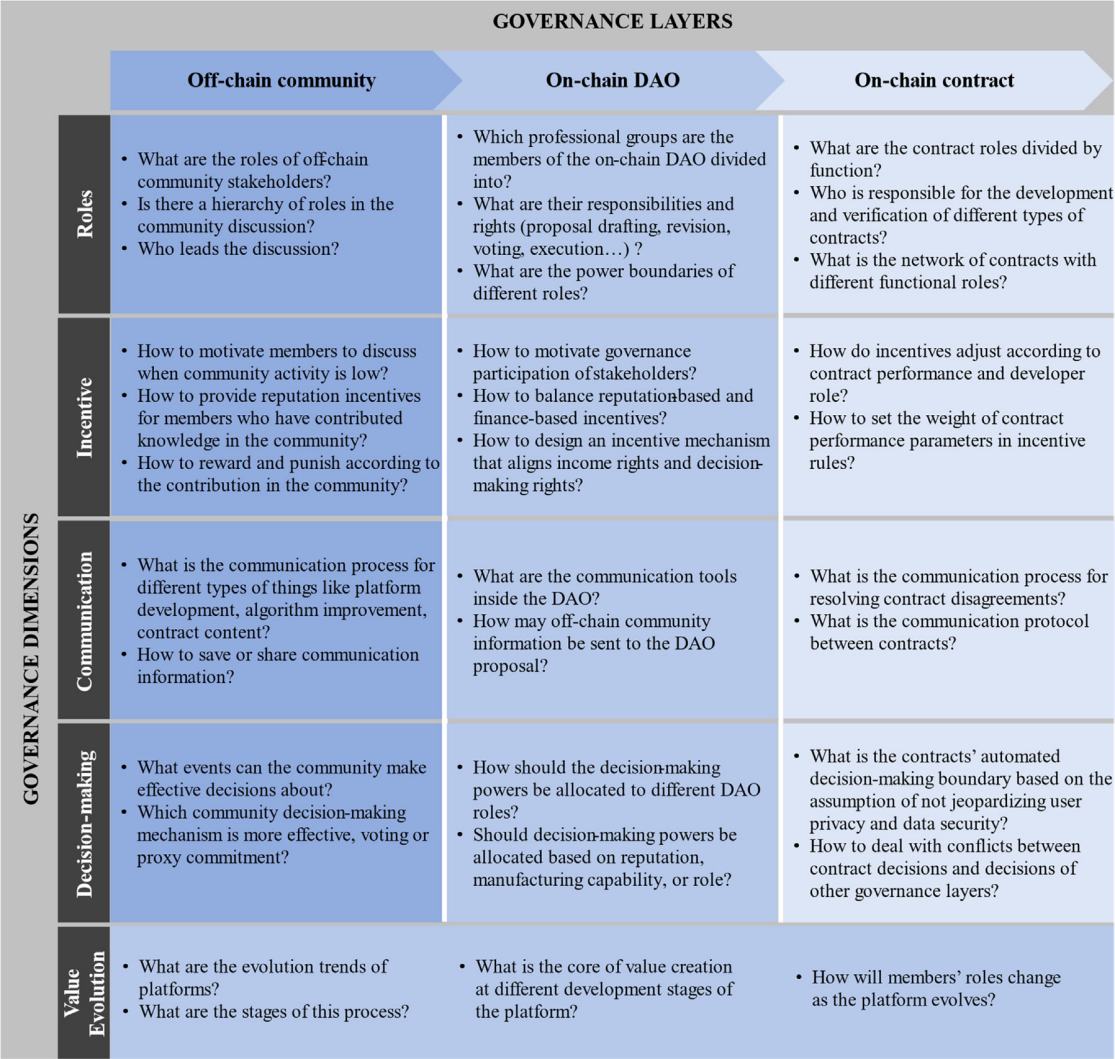
**The governance framework of BMCP**.

Drawing from the four dimensions of previous platform governance research and the “value evolution” characteristic of DAOs, while considering the three-tier governance structure established in DAO-related literature, this paper constructs a five-dimensional, three-level BMCP governance framework (Fig. 2). This framework aims to comprehensively guide BMCP Governance practices across different layers and dimensions.

### 4.1 Governance dimensions

**Fig. 3:**
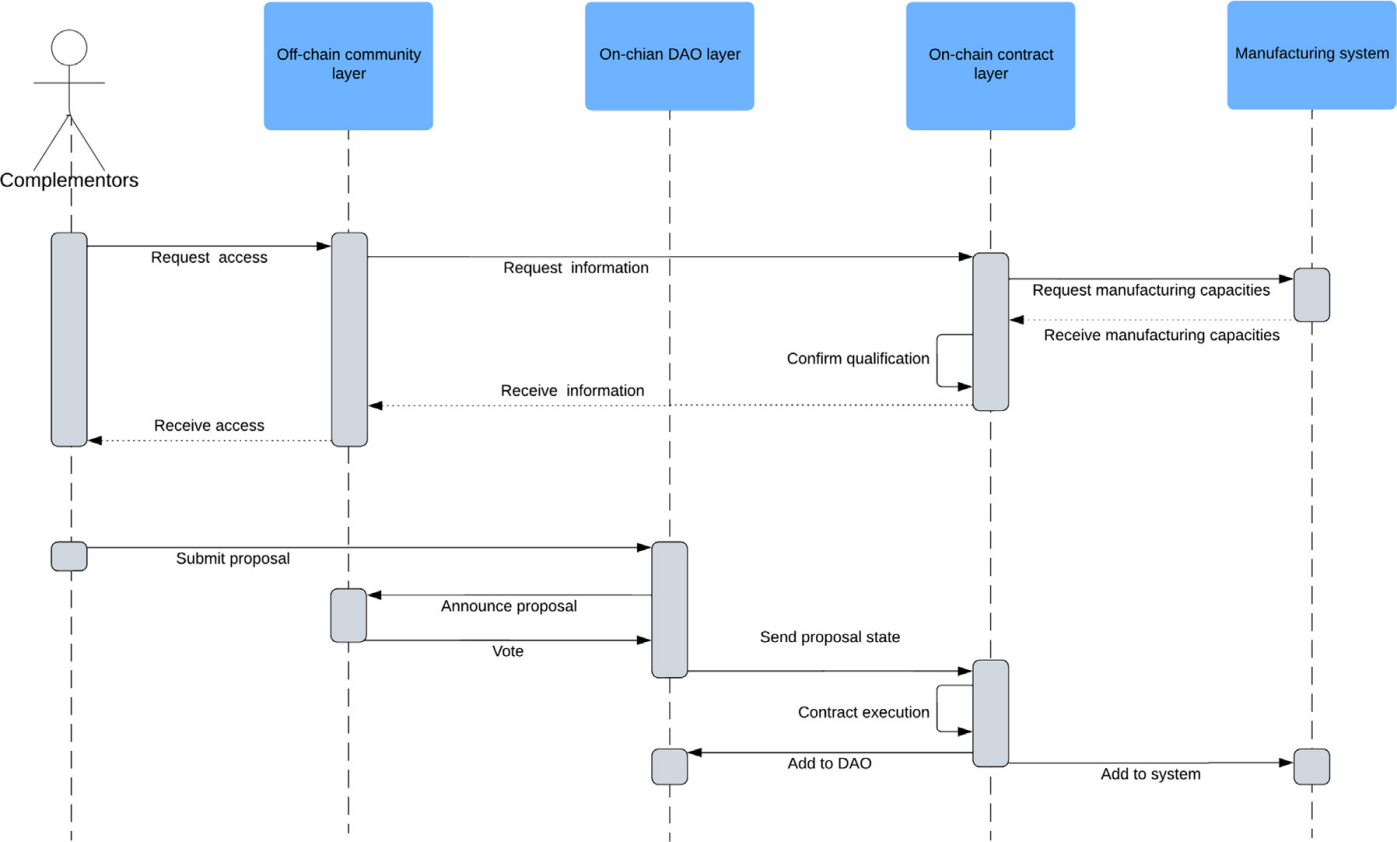
**Member expansion decision process**.

**Fig. 4:**
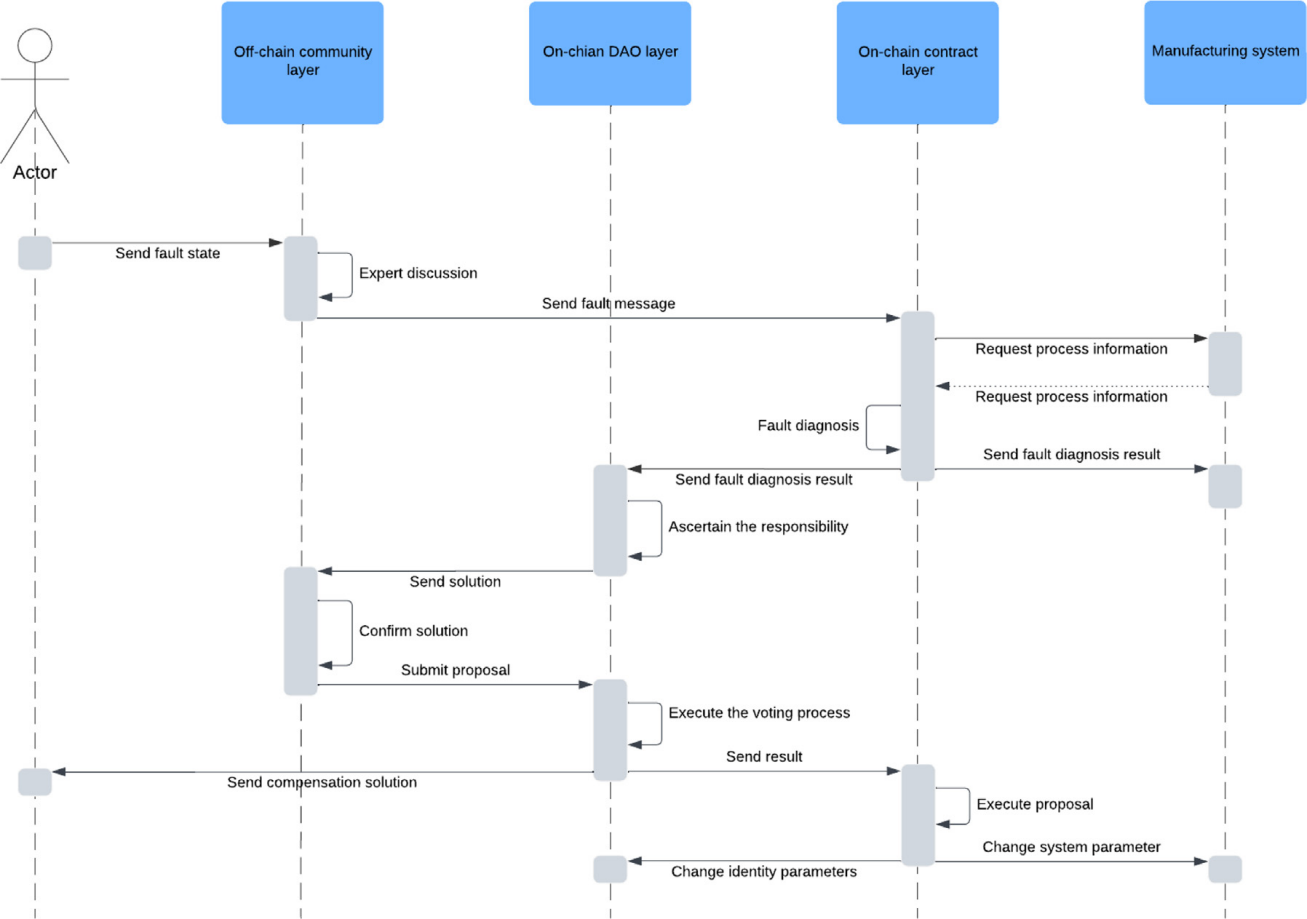
**Fault management process**.

From the perspective of vertical governance dimensions, the BMCP governance framework designed in this paper includes the traditional dimensions of roles, incentives, communication, and decision-making, and innovatively introduces the “value evolution” dimension [50]. Each dimension carries unique functions and importance, detailed as follows:

Roles dimension: This dimension focuses on defining the identities of different stakeholders within the three-tier governance structure, including platform operators, manufacturers, and service providers. It emphasizes clarifying the responsibilities and obligations of each role, designing appropriate access permissions, and standardizing interactions between different levels to ensure the orderly operation of the platform ecosystem (Fig. 3, Fig. 4).

Incentives dimension: This dimension aims to design incentive mechanisms based on the intrinsic motivations of various roles within the BMCP, with the goal of stimulating active participation from all stakeholders and maintaining the platform's long-term stability and vitality. Through carefully designed incentive strategies, the platform can attract and retain valuable participants, fostering a prosperous ecosystem.

Communication dimension: This dimension addresses information exchange and consensus formation among BMCP stakeholders, encompassing the use of formal and informal communication tools such as chat tools, coordination systems, recording systems, regular public discussions, and specialized group meetings. These tools promote transparent information flow and the aggregation of collective wisdom.

Decision-making dimension: This dimension involves the integration of information and execution of tasks at different governance levels, emphasizing the design of collective decision-making mechanisms within DAOs. These include voting, information dissemination, consensus-building, and conflict resolution procedures, ensuring efficient and fair decision-making processes that adapt to the dynamic needs of various business scenarios [77].

Value evolution dimension: This dimension highlights the unique aspect of BMCP compared to traditional platform governance. It focuses on how the decentralized nature of the manufacturing service platform drives the evolution of platform value over time, reflecting trends in core functions, stakeholder motivations, resource quality, and the roles of core members at different stages of development. Blockchain-based decentralization results in faster and more uncertain value evolution, necessitating the consideration of dynamic value evolution when designing governance mechanisms to achieve continuous optimization and adaptive growth of the platform ecosystem [29].

### 4.2 Governance layers

The external interference of the platform ecosystem can be divided into several levels according to the size of the influence, including the machine level, the operational level, the enterprise level, the market level, and the social level [71]. Therefore, the governance structure of BMCP also needs to distinguish levels on the basis of flatness, so as to balance efficiency and fairness when dealing with different levels of risks. From the perspective of horizontal governance levels, the governance framework designed for BMCP in this paper consists of three layers: the off-chain community layer, the on-chain DAO layer, and the on-chain contract layer [10,78]. The specific details are as follows:

Off-chain community layer: This layer directly interfaces with the real world, managing affairs closely tied to everyday life. The core tasks of the off-chain community layer include thoroughly explaining the operational mechanisms and rule systems of BMCP to ensure that community members fully understand and accept these rules. It also facilitates the flow of information between the community and the other governance levels on the blockchain. The governance challenge at this level lies in efficiently collecting and analyzing complex information flows within the community and transforming them into actionable decision-making bases, thereby maintaining the health and sustainable development of the community [79,80].

On-chain DAO layer: This layer serves as a digital extension of the off-chain community, achieving automation and democratization of governance through the use of smart contracts. The on-chain DAO layer focuses on key areas such as value distribution and interest coordination, ensuring that the value generated within the manufacturing chain is fairly and promptly distributed among stakeholders. Specific tasks include reviewing and voting on community proposals, implementing voting results, and providing automated solutions for potential conflicts of interest. The governance focus at this level is on designing secure smart contracts, ensuring a reasonable allocation of decision-making power, and standardizing the proposal process to ensure smooth and efficient governance activities.

On-chain contract layer: As the execution terminal of the governance framework, this layer is responsible for translating the decisions of the previous two layers into specific blockchain operations. It handles technical implementations such as adjusting blockchain network parameters, setting up voting mechanisms, and managing user permissions. This layer ensures that all decisions are accurately executed within the blockchain environment, maintaining the platform's stability and security. The challenge at this level is to continuously optimize technical details to adapt to rapidly changing business demands and external environments, while ensuring the platform's long-term scalability.

Through the synergistic effect of these three layers, BMCP constructs an efficient governance framework that can respond to real-world needs while fully leveraging the advantages of blockchain technology. This framework achieves comprehensive coverage from macro policy to micro execution.

### 4.3 Discussion: Combining layers and dimensions

Although the governance levels of BMCP exhibit a logical progression, this sequence is not fixed and can be flexibly adjusted based on specific scenarios in practice. For instance, when disputes among stakeholders can be swiftly resolved through off-chain communication without escalating to the on-chain DAO layer, agreements can be executed directly at the on-chain contract layer. Therefore, the framework must be applied flexibly in different governance contexts to achieve optimal results. Next, we will discuss the flexible integration of layers and dimensions.

Off-chain community layer: In the role dimension, it is essential to clearly define the responsibilities of different stakeholders, especially during proposal discussions, by assigning leadership and collaborative roles to ensure an efficient and orderly decision-making process [81]. In the incentive dimension, the off-chain community layer must implement reward and punishment mechanisms to maintain information quality, sustain member engagement, and invigorate the community. The communication dimension requires defining the communication processes among stakeholders for various collaborative tasks and providing tools to verify information authenticity, ensuring decentralized communication's efficiency and consensus. The decision dimension involves clarifying power boundaries, specifying which decisions apply to which events, and ensuring the reasonableness and fairness of decisions to avoid power abuse. The value evolution dimension, with its long-term orientation, influences the community's development direction, ensuring alignment between platform values and community goals.

On-chain DAO layer: In the role dimension, it is crucial to meticulously design the operational processes for professional groups, ensuring that each group's permissions and member management mechanisms align with the enforcement power of smart contracts, laying the foundation for the orderly operation of the platform ecosystem. In the incentive dimension, the on-chain DAO layer incorporates governance behaviors like voting into reputation algorithms and designs incentive mechanisms to effectively stimulate user participation in governance activities when engagement is low, fostering community activity and consensus building. In the communication dimension, the on-chain DAO layer establishes seamless communication mechanisms with the off-chain community, ensuring traceability and verification of decision-related information through trusted on-chain and off-chain interactions, maintaining transparency and accuracy in information transmission. The decision dimension focuses on designing power distribution algorithms, using multidimensional data such as community performance, manufacturing capacity, and transaction information to adjust algorithms according to different proposal types, preventing imbalances between decision-making and profit rights, and ensuring fair and reasonable power distribution. The value evolution dimension also has a long-term impact on the design of decision-making and incentive mechanisms, promoting continuous optimization of the BMCP ecosystem.

On-chain contract layer: In the role dimension, it is necessary to clearly classify the functions of smart contracts to avoid potential network security risks and ensure reliable contract execution. In the incentive dimension, given the severe consequences of contract vulnerabilities, designing incentive mechanisms to reward excellent contract designers becomes crucial, ensuring work quality is a consideration in decision-making power distribution, promoting the specialization and security of contract design. In the communication dimension, the on-chain contract layer must provide diverse communication tools, both formal and informal, to help stakeholders reach consensus when there are disagreements about contract content, reinforcing the negotiation and revision process of contracts. Additionally, to avoid execution obstacles, standardizing communication methods between different developers' contracts is necessary to ensure consistency and interoperability between contracts. The decision dimension focuses on clarifying the boundaries of smart contract automatic execution, especially in scenarios where contract content conflicts with higher-level decisions, defining execution authority to avoid potential governance conflicts and ensure consistency and coherence of platform rules. The value evolution dimension guides the design and optimization of smart contracts, ensuring the synchronization of contract functions with platform values and laying a solid foundation for the continuous innovation and development of BMCP.

Through the synergistic effect of the five dimensions—role, incentive, communication, decision, and value evolution—and the three governance layers—off-chain community layer, on-chain DAO layer, and on-chain contract layer—the BMCP governance framework demonstrates its unique advantages and significance. The comprehensive application of the five dimensions ensures the governance mechanism's thoroughness and flexibility, enhancing collaboration efficiency and trust foundation within the platform ecosystem while encouraging active participation and value co-creation among members. The progressive advancement of the three layers constructs a multidimensional governance system from the bottom up and from the outside in, achieving full-chain coverage from community building to smart contract execution, ensuring decision fairness and execution efficiency. This framework fully leverages blockchain technology's potential, combining smart contracts and DAOs to address the centralization bottlenecks and trust issues in traditional platform governance, providing a secure and credible digital infrastructure for manufacturing collaboration. The introduction of the value evolution dimension also enables BMCP to adapt to market changes and technological advancements, continuously optimizing the platform ecosystem. Therefore, the BMCP governance framework proposed in this paper not only provides an innovative paradigm for platform governance but also lays a solid institutional foundation for building an open, inclusive, and shared new global manufacturing ecosystem.

## 5 Two-stage BMCP governance solutions

Due to the openness, uncertainty and complexity of BMCP, the governance priorities of BMCP in different development stages have changed significantly [25,73,82]. In this section, we will analyze the two different stages of BMCP from the perspective of platform value evolution, and use UML sequence diagrams to illustrate how the governance framework handles different issues. This method is often used in BMCP research to verify collaborative processes and platform framework design [45,83,84].

### 5.1 Stage 1: Start-up stage

The start-up stage is the first stage of platform development. This stage of governance consists of two components: platform establishment and membership extension. The goals of governance in this stage are setting the basic rules and values of the BMCPs, and rapidly expanding the value chain to maximize the network effect.

When governing the platform establishment, the project leader first needs to clarify the core functions and target groups of the platform, which determines the business model. Trust relationships based on consensus mechanisms and secure traceable data are the core competitiveness of the BMCPs. Therefore, in addition to manufacturing resource order matching, the main profit methods of the platform should include data management services, infrastructure services for developers, and solutions to improve customer satisfaction. The development of core functions of the platform and the expansion of target groups should be carried out around these services. Second, the BMCPs need to become a legal entity. Becoming a legitimate platform is the prerequisite for attracting all parties to participate in the collaboration. On this basis, platform rules are feasible for automatic execution in terms of encouraging business innovation, improving market penetration, and establishing standardized processes. This external legitimacy provides ecological constraints to ensure high service quality at the start-up stage. Finally, the platform start-up team needs to investigate potential platform complementors through the off-chain community and identify the types of core stakeholders at an early stage, such as manufacturing service providers, on-chain and off-chain information flow managers, registrars providing identity authentication, standardization organizations that design standard contracts, and professional governance organizations that provide proposal drafting services.

When governing the membership expansion, due to the uneven quality of manufacturing resources and capabilities in BMCPs at this stage, potential stakeholders are affected by the uncertainty of risks and take a wait-and-see attitude. Therefore, the goal of platform governance is to ensure that the value proposition of the platform is seen and understood by more external users to attract more third-party complementors. This requires scientific promotion strategies and membership access mechanisms. When formulating a promotion strategy, it is necessary to clarify the non-profit position of BMCPs in the start-up stage and open it to members who are consistent with the platform’s values, to attract early stakeholders. In addition, BMCPs should give priority to encouraging well-known manufacturers to join and use their brand effects to attract the attention of the media and their original supply chain to promote the participation of upstream and downstream members. When formulating the membership access mechanism, the supply chain partners of the initial users in the industrial B2B platforms have a strong motivation to participate [85]. The invitation guarantee mechanism can not only attract users from different industry chains to participate but also ensure the consistency of interests. This helps to lower operating costs during a period when governance mechanisms are imperfect. It needs to be emphasized that DAO should write member access conditions into smart contracts through on-chain voting to maintain the long-term quality level of members.

According to the proposed governance framework, we use UML sequence diagrams to detail the decision process of member expansion (Fig. 3). The design of the member expansion process is the key to the success of BMCP crossing the start-up stage. It is directly related to the openness, complexity and uncertainty governance of the platform, which determines the available scope of platform resources and the cost of ecological governance [[86], [87], [88], [89]]. First, the platform complementor makes resource access requests to the off-chain community layer. The community publicizes the basic information of the applicant (industry, scale, location, product details, etc.), and selects candidates according to multi-dimensional indicators such as discussion heat, platform ecological needs and feedback from members. The off-chain community then requests information about the candidate's manufacturing capabilities from the on-chain contract. The on-chain contract automatically executes and sends a request to the manufacturing system to obtain information about the candidate's manufacturing capabilities (type of equipment, number of equipment, capacity, downtime, etc.). The contract layer that receives the feedback calls the contract to verify the authenticity of the candidate's information and whether it meets the platform entry threshold. After the on-chain contract confirms the candidate's eligibility, the processed information is transmitted to the off-chain community layer. The off-chain community layer sends permission information to the complementor (access rights vary for different types of complementor). After the complementor is authorized, it needs to submit a proposal to the on-chain DAO layer, and the proposal needs to contain relevant information. The content of the proposal can be divided into necessary information and custom information, standardized by contracts in the on-chain DAO. The on-chain DAO layer publicizes the proposal information to the community and reminds stakeholders to participate in the voting within the specified time. After the proposal result is sent to the on-chain contract layer, the contract automatically executes the details in the approved proposal. Finally, depending on the result of the contract execution, the manufacturing system and the on-chain DAO layer modify the internal parameters, including the permission code of the complementor, reputation score and resource pool number.

In a word, platform governance during the start-up stage should focus on self-positioning and value enhancement. The platform ecosystem during this stage is characterized by welfare, interest consistency, and active openness. BMCP governance is mainly based on the off-chain community layer, supplemented by the on-chain DAO layer. Governance mainly consists of roles, incentives, and communication governance dimensions. By defining potential participation roles, motivating individuals to participate, and providing formal or informal communication tools for community discussions, BMCP can successfully move beyond the early stages.

### 5.2 Stage 2: Optimization stage

Following the start-up stage, the platform ecosystem becomes diverse and enters a stage of rapid development driven by network effects. The explosion of complex stakeholders and the continuous change of business service present new challenges to the governance of group behavior and rights rules in BMCPs. Therefore, governance at this stage should focus on group behavior and rule optimization.

The connotation of group behavior governance is to coordinate complex cooperation and competition behaviors. Group behavior in BMCPs has two characteristics. On the one hand, competition and collaboration can be balanced in complex, interconnected environments [86]. While there may be competition in business interactions, the value created between manufacturers by integrating complementary or similar resources exceeds that created by working alone [[87], [90]]. On the other hand, unlike the winner-take-all situation in B2C, the high cost of manufacturing resources and required expertise in B2B scenarios lead to frequent changes in cooperation and competition states [88,[89], [91], [92]]. Members collaborate in some activities while competing in others to spur innovation. Fig. 4 illustrates how the governance framework is applied to BMCP group behavior governance using fault management in manufacturing collaboration as an example. Fault management is a series of governance solutions to control members' misconduct and manufacturing system failure rates. It requires the support of multi-dimensional governance mechanisms of incentives, decision-making, communication and roles to reduce systemic risks [[93], [94], [95], [96]].

First, the actors (co-manufacturers, consumers, third-party platform complementors) who find the fault of the co-manufacturing product send the fault judgment basis to the off-chain community layer. Subsequently, experts in the off-chain community layer combine experience and knowledge to discuss and confirm the fault. This step is designed to filter out noise to reduce the burden on the on-chain governance layer and avoid the risk of irreversible decisions. When the off-chain community confirms the fault status, information is sent to the on-chain contract requesting diagnosis. On-chain contracts request manufacturing process data from manufacturing systems and perform troubleshooting processes. After identifying the failure factor, the contract automatically sends a signal to the manufacturing system for fault warning, while sending diagnostic results to the on-chain DAO layer to support accountability. The on-chain DAO layer automatically generates a fault solution according to the rules of the consensus contract between members, including value redistribution, permission change, credit score adjustment and other measures. The proposal is sent to the off-chain community for full discussion. After confirmation, the community drafts a normative proposal based on the solution and submits it to the on-chain DAO for democratic decision-making. After the on-chain DAO layer executes the decision process, it compensates the actor based on the solution content and synchronizes the state to the on-chain contract layer. The on-chain contract layer executes governance contracts based on the proposal results to further optimize the platform ecosystem. On the one hand, the contract will reset the identity management system in the on-chain DAO, including decision rights, resource access restrictions, reputation scores, and collaboration levels. On the other hand, the contract will modify parameters in the manufacturing system, such as equipment priority, failure risk index, maintenance cycle and resource value.

Tokenization technology based on the blockchain can efficiently manage group behavior. It enables innovators to create digital tokens to represent scarce assets, potentially changing the innovation scene [97]. In BMCPs, tokens can be used to manage value creation and transfer within a collaboration network. Tokenization of manufacturing resources associates the token value with the manufacturing resources, and then the value generated by the full lifecycle service is linked to the token value. On the one hand, it can improve the alignment of stakeholder value with platform value. On the other hand, it helps to decentralize the responsibility of service providers in the composite tasks and realize the consistency of responsibility and rights in multiple parties’ participation. Furthermore, value contribution within the collaboration network becomes transparent, which might inspire higher-quality innovation. When tokens are linked to DAO governance rights, platform decisions can be consistent with key members, which is beneficial to the platform’s long-term development.

The core of rule optimization is to delineate clear boundaries of rights. Because data storage and exchange services in BMCPs are important competitive advantages. Among the core rights of platform stakeholders, those related to data include ownership of production data, hierarchical data access rights, data control rights, and data governance rights. Therefore, one of the purposes of rule optimization is to encourage users to share data more openly to stimulate data-based service innovation. Smart contracts have the advantages of transparency and reliability in defining rights rules and can reduce members’ exposure to private data when automatically executing rules. For example, changes to data management contract codes are decided by DAO members and can be executed automatically after the contract is deployed without the need for managers to access the data.

User management is an important part of rule optimization. On the one hand, it is necessary to consider the service quality of different service providers to dynamically adjust their power boundaries. For example, Alibaba Cloud’s selection mechanism encourages users at different levels to participate by signing hierarchical support agreements and promoting high-quality innovation activities [76]. On the other hand, the operator’s power boundary is also part of user management. The role of platform operators will gradually shift from monitoring and control to optimizing collaboration processes and providing a user-centric independent development environment. Therefore, the resources they are allowed to use also need to be first discussed by the off-chain community, then voted on by the on-chain DAO, and finally written into the on-chain contract.

In short, the key governance issues in the optimization stage are ensuring group behavior consistent with the long-term value of BMCPs, and using smart contracts to manage member rights. At this stage, the platform ecosystem presents the characteristics of complex interests and uncertain behaviors. The on-chain contracts layer is crucial in this stage of BMCP governance. The main governance dimensions are incentives and decision-making.

## 6 Conclusions and future work

This paper addresses the limitations of traditional MCPs, such as trust deficits and innovation suppression, which have become more pronounced in the face of globalization. It explores BMCPs as a potential solution for these issues. Given the BMCP’s characteristics of openness, uncertainty, and complexity, the paper first systematically analyzes the similarities and differences between MCP and BMCP ecosystems. Guided by these findings, it identifies six core governance challenges for BMCPs: platform positioning, value goal alignment, necessity of hierarchical structures, trust mechanism construction, balancing openness and stability, and decision-making mechanism design. The paper then innovatively proposes a five-dimensional, three-layer BMCP governance framework. This framework is designed to provide comprehensive and flexible guidance for BMCP governance practices across five dimensions—roles, incentives, communication, decision-making, and value evolution—and three governance layers: the off-chain community layer, the on-chain DAO layer, and the on-chain contract layer. By elaborating on the principles and application strategies of this framework, the paper demonstrates how to flexibly apply it in various governance scenarios to address the dynamic changes and complex demands of the BMCP ecosystem. Finally, considering the value evolution characteristic of BMCPs, the paper further explores how to effectively apply the governance framework during the initial and optimization stages of platform development. With the help of intuitive diagrams, it presents a visual path of BMCP governance evolution for better understanding and practical implementation. This paper not only contributes a theoretical framework and practical guidelines to the BMCP governance research field but also offers profound insights into the future development direction of manufacturing platforms. It aims to promote the manufacturing ecosystem towards greater openness, intelligence, and collaboration.

Although BMCPs have shown initial success in certain industries, their application scope and impact need further expansion. Current research is still in its early stages, and many areas remain unexplored. This paper proposes six research directions for future study:

(1) Decision-making power allocation mechanism for manufacturing collaboration alliance members. In the BMCP ecosystem, a key issue is how to fairly and efficiently allocate decision-making power among various stakeholders. Future research should delve into the design of algorithms for power allocation, considering factors such as contribution, trust, and activity levels. Additionally, dynamic adjustment mechanisms should be explored to adapt to changes in alliance membership and the evolution of the platform ecosystem.
(2) Design of dynamic layered user management mechanisms. As the BMCP platform grows, the number and types of users will increase. Designing a flexible layered user management system that meets the needs of different user groups while ensuring efficient and secure platform operations will be a significant research focus. This includes setting standards for user classification, developing access control strategies, and implementing automated management through smart contracts.
(3) Exploration of applicable industries and business models for BMCP. Currently, BMCP applications are primarily concentrated in certain segments of the manufacturing industry. Future research should broaden its scope to explore the potential of BMCPs in other industries such as energy, healthcare, and logistics. Additionally, identifying innovative business models that match these new applications will be crucial for fostering cross-industry collaborative innovation and value creation.
(4) Design of internal platform rules consistent with external regulations. Ensuring that platform rules comply with external laws, industry standards, and ethical guidelines is essential for the legal and compliant operation of BMCPs. Future research should focus on designing a rule system that adheres to external regulations while meeting internal governance needs, ensuring the healthy development of the BMCP ecosystem.
(5) Interaction models between platform operators and complementary service providers. The cooperation between platform operators and complementary service providers directly impacts the diversity and vitality of the BMCP ecosystem. Research should focus on creating mutually beneficial cooperation mechanisms, including profit distribution, resource sharing, and risk-sharing, to promote ecosystem diversity and sustainable development.
(6) Participation of integrators, designers, and other service providers. The roles and contributions of integrators, designers, and other service providers within the BMCP ecosystem warrant in-depth investigation. Future research should analyze how these participants integrate into the platform, how their services complement existing resources, and how to design incentive mechanisms to encourage their long-term participation and value contribution.

## Declaration of competing interest

The authors declare that they have no conflicts of interest in this work.

## Footnotes

1 https://www.mckinsey.com/capabilities/operations/our-insights/risk-resilience-and-rebalancing-in-global-value-chains.
